# Supplementary material for: An efficient method to clone TAL effector genes from Xanthomonas oryzae using Gibson assembly
Source: Mol Plant Pathol. 2019 Aug 15;20(10):1453–62. doi: 10.1111/mpp.12820 (PMC6792135; doi:10.1111/mpp.12820)
Supplement: Supplementary file 1 — Fig. S1 DNA sequences of gBlock fragments synthesized to make two Gibson cloning vectors. (A) gBlock1 was used to inserted into pZW‐pthXo1 at SphI sites through Gibson cloning. The two sequences shaded in yellow are homologous to the ends of SphI fragments of TALe genes. (B) gBlock2 was used to make pHM1‐Gib. The two sequences shaded in yellow are homologous to the ends of BamHI fragments of TALe genes. [file MPP-20-1453-s001.docx]

**gBlock1 sequence for pZW-Gib**

*Sph*I

**A**

*Bsm*BI

GGCGTGACCGCAGTGGAGGCAGT***GCATGC***ATGGCGCAATGCACTGACGGGTGCA***GAGACG***ATAACAGTATGCGTATTTGCGCGCTGATTTTTGCGGTATAAGAATATATACTGATATGTATACCCGAAGTATGTCAAAAAGAGGTATGCTATGAAGCAGCGTATTACAGTGACAGTTGACAGCGACAGCTATCAGTTGCTCAAGGCATATATGATGTCAATATCTCCGGTCTGGTAAGCACAACCATGCAGAATGAAGCCCGTCGTCTGCGTGCCGAACGCTGGAAAGCGGAAAATCAGGAAGGGATGGCTGAGGTCGCCCGGTTTATTGAAATGAACGGCTCTTTTGCTGACGAGAACAGGGGCTGGTGAAATGCAGTTTAAGGTTTACACCTATAAAAGAGAGAGCCGTTATCGTCTGTTTGTGGATGTACAGAGTGATATTATTGACACGCCCGGGCGACGGATGGTGATCCCCCTGGCCAGTGCACGTCTGCTGTCAGATAAAGTCTCCCGTGAACTTTACCCGGTGGTGCATATCGGGGATGAAAGCTGGCGCATGATGACCACCGATATGGCCAGTGTGCCGGTCTCCGTTATCGGGGAAGAAGTGGCTGATCTCAGCCACCGCGAAAATGACATCAAAAACGCCATTAACCTGATGTTCTGGGGAATATAATGAGGCTCCCTTATACAC***CGTCTC***AACGCCGGATCAGGCGTCTTT***GCATGC***ATTCGCCGATTCGCTGGAG

*Bsm*BI

*Sph*I

**B**

**gBlock2 sequence for pHM1-Gib**:

*Hind*III

CACGCCAAGTCCTGCCCGCG***AAGCTT***GCATTAGGCACCCCAGGCTTTACACTTTATGCTTCCGGCTCGTATAATGTGTGGATTTTGAGTTAGGATCGATCCGGCTTACTAAAAGCCAGATAACAGTATGCGTATTTGCGCGCTGATTTTTGCGGTATAAGAATATATACTGATATGTATACCCGAAGTATGTCAAAAAGAGGTATGCTATGAAGCAGCGTATTACAGTGACAGTTGACAGCGACAGCTATCAGTTGCTCAAGGCATATATGATGTCAATATCTCCGGTCTGGTAAGCACAACCATGCAGAATGAAGCCCGTCGTCTGCGTGCCGAACGCTGGAAAGCGGAAAATCAGGAAGGGATGGCTGAGGTCGCCCGGTTTATTGAAATGAACGGCTCTTTTGCTGACGAGAACAGGGGCTGGTGAAATGCAGTTTAAGGTTTACACCTATAAAAGAGAGAGCCGTTATCGTCTGTTTGTGGATGTACAGAGTGATATTATTGACACGCCCGGGCGACGGATGGTGATCCCCCTGGCCAGTGCACGTCTGCTGTCAGATAAAGTCTCCCGTGAACTTTACCCGGTGGTGCATATCGGGGATGAAAGCTGGCGCATGATGACCACCGATATGGCCAGTGTGCCGGTCTCCGTTATCGGGGAAGAAGTGGCTGATCTCAGCCACCGCGAAAATGACATCAAAAACGCCATTAACCTGATGTTCTGGGGAATATAAATGTCAGGCTCCCTTATACACAGCCAGTCTGCAGGTCGA***AAGCTT***ACCAGGATCGGGGGCGGCCT

*Hind*III

**Supplementary Fig. S1.** DNA sequences of gBlock fragments synthesized to make two Gibson cloning vectors. A. gBlock1 was used to inserted into pZW-pthXo1 at *Sph*I sites through Gibson cloning. The two sequences shaded in yellow color are homologous to the ends of *Sph*I fragments of TALe genes. B. gBlock2 was used to make pHM1-Gib. The two sequences shaded in yellow color are homologous to the ends of *Bam*HI fragments of TALe genes.
