# Supplementary material for: An efficient method to clone TAL effector genes from Xanthomonas oryzae using Gibson assembly
Source: Mol Plant Pathol. 2019 Aug 15;20(10):1453–62. doi: 10.1111/mpp.12820 (PMC6792135; doi:10.1111/mpp.12820)
Supplement: Supplementary file 2 — Fig. S2 Nine TALe genes from AXO1947 were cloned using the pZW‐Gib vector and Gibson assembly method. The RVDs of individual TALes are shown under numbers 1 to 26, indicating the order of 33–34 amino acid repeats. Asterisks (*) indicate that the amino acid at the 13th position missing. [file MPP-20-1453-s002.docx]

**
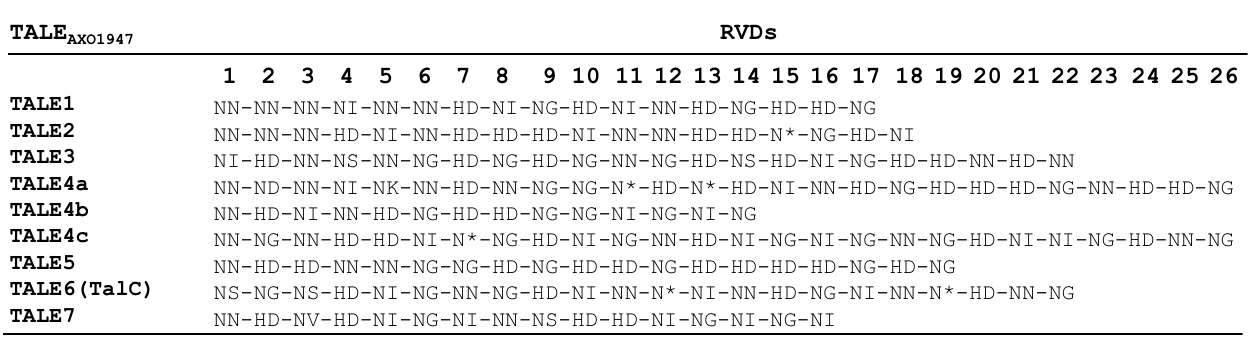
Supplementary Fig. S2** Nine TALe genes from AXO1947 were cloned using pZW-Gib vector and Gibson assembly method. The RVDs of individual TALes are shown under numbers (1 to 26) indicating the order of 33-34 amino acid repeats. Asterisk (*) indicates amino acid at the 13^th^ position missing.
