# Supplementary material for: An efficient method to clone TAL effector genes from Xanthomonas oryzae using Gibson assembly
Source: Mol Plant Pathol. 2019 Aug 15;20(10):1453–62. doi: 10.1111/mpp.12820 (PMC6792135; doi:10.1111/mpp.12820)
Supplement: Supplementary file 3 — Fig. S3 Virulence contribution of nine TALes cloned from AXO1947. (A) Lesion lengths caused in rice Kitaake and Zhenshan 97 (ZS 97) by different Xoo strains indicated below the paired columns. Different lower letters indicate statistically significant differences (mean SEM, n = 10, P < 0.05). (B) Blight symptom in Kitaake leaves caused by the Xoo strains as indicated below each leaf. Arrows indicate the edges of lesions. [file MPP-20-1453-s003.docx]

**
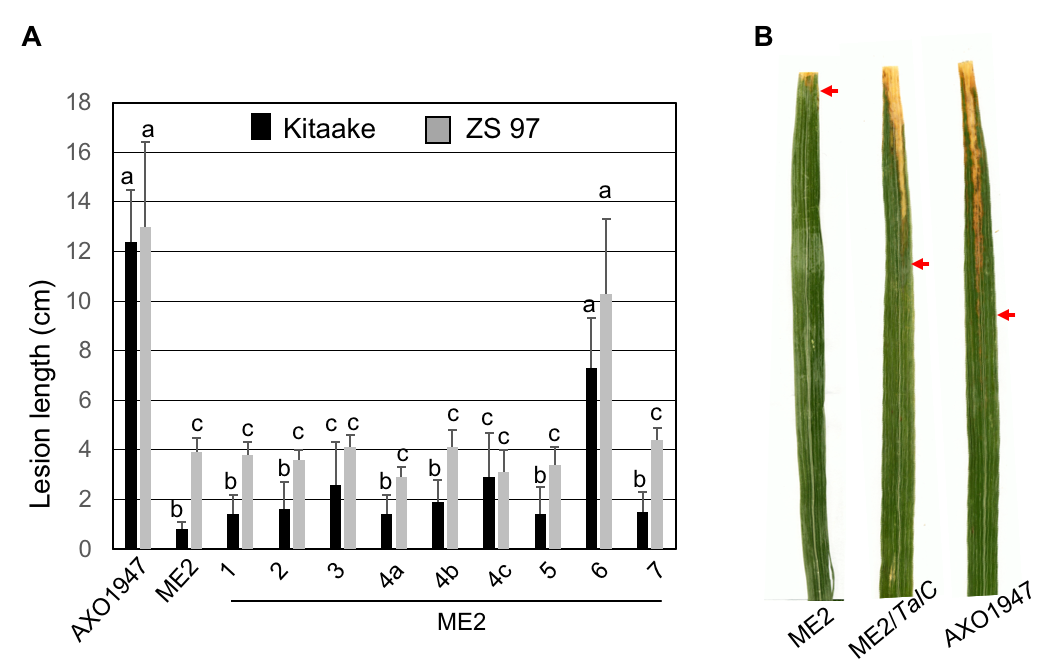
**

**Supplementary Fig. S3** Virulence contribution of nine TALes cloned from AXO1947. **A.** Lesion lengths caused in rice Kitaake and Zhenshan 97 (ZS 97) by different Xoo strains indicated below the paired columns. Different lower letters indicate statistically significant difference (means ± s.e.m, n=10, *P*<0.05). **B.** blight symptom in Kitaake leaves caused by the Xoo strains as indicated below each leaf. Arrow indicates the edge of lesion.
