# Supplementary material for: An efficient method to clone TAL effector genes from Xanthomonas oryzae using Gibson assembly
Source: Mol Plant Pathol. 2019 Aug 15;20(10):1453–62. doi: 10.1111/mpp.12820 (PMC6792135; doi:10.1111/mpp.12820)
Supplement: Supplementary file 4 — Fig. S4 TALe genes from PXO61 and AXO1947. (A) TALe genes clustered in the genome of PXO61. (B) TALe genes clustered in the genome of AXO1947. (C, D) Lesion length measurements (mean SEM, n = 10) in Kitaake caused by different strains. [file MPP-20-1453-s004.docx]

**
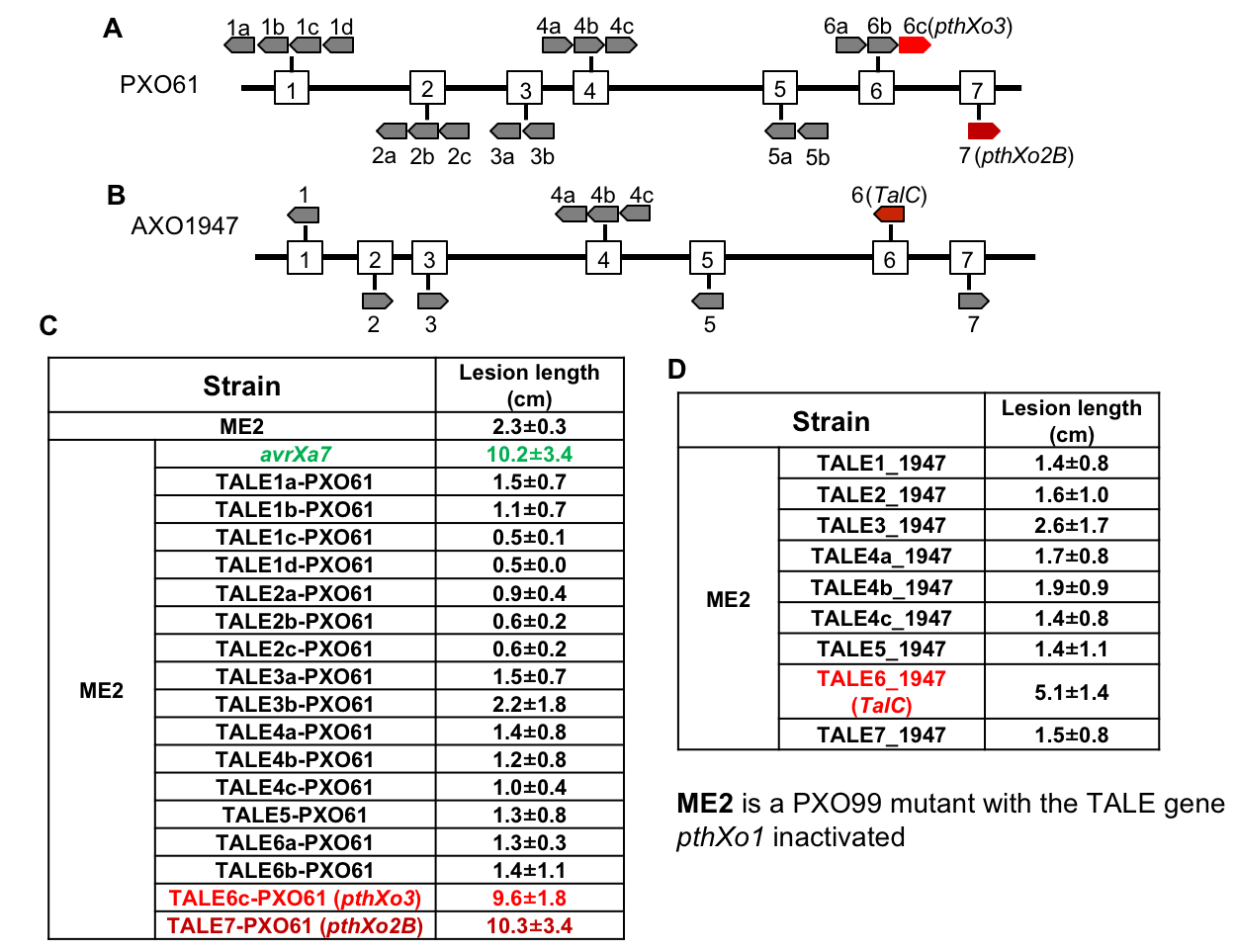
**

**Supplementary Fig. S4** TALe genes from PXO61 and AXO1947. **A.** TALe genes clustered in the genome of PXO61. **B**. TALe genes clustered in the genome of AXO1947. **C**, **D**. Lesion length measurements (mean ± s.e.m, n=10) in Kitaake caused by different strains.
