# Supplementary material for: An efficient method to clone TAL effector genes from Xanthomonas oryzae using Gibson assembly
Source: Mol Plant Pathol. 2019 Aug 15;20(10):1453–62. doi: 10.1111/mpp.12820 (PMC6792135; doi:10.1111/mpp.12820)
Supplement: Supplementary file 6 — Fig. S6 Validation of TALe clones through PCR, restriction enzyme digestions. (A) Schematics of selective isolation of BamHI fragments TALe genes from genomic DNA of CFBP7325. (B) Validation of TALe clones through PCR with primers P‐F1 and P‐R1 of individual clones as indicated above lanes of upper gel image, digestion by MscI which cuts each of central repeats (the DNA band patterns resulted from partial digestion) and digestion by BamHI. [file MPP-20-1453-s006.docx]

**
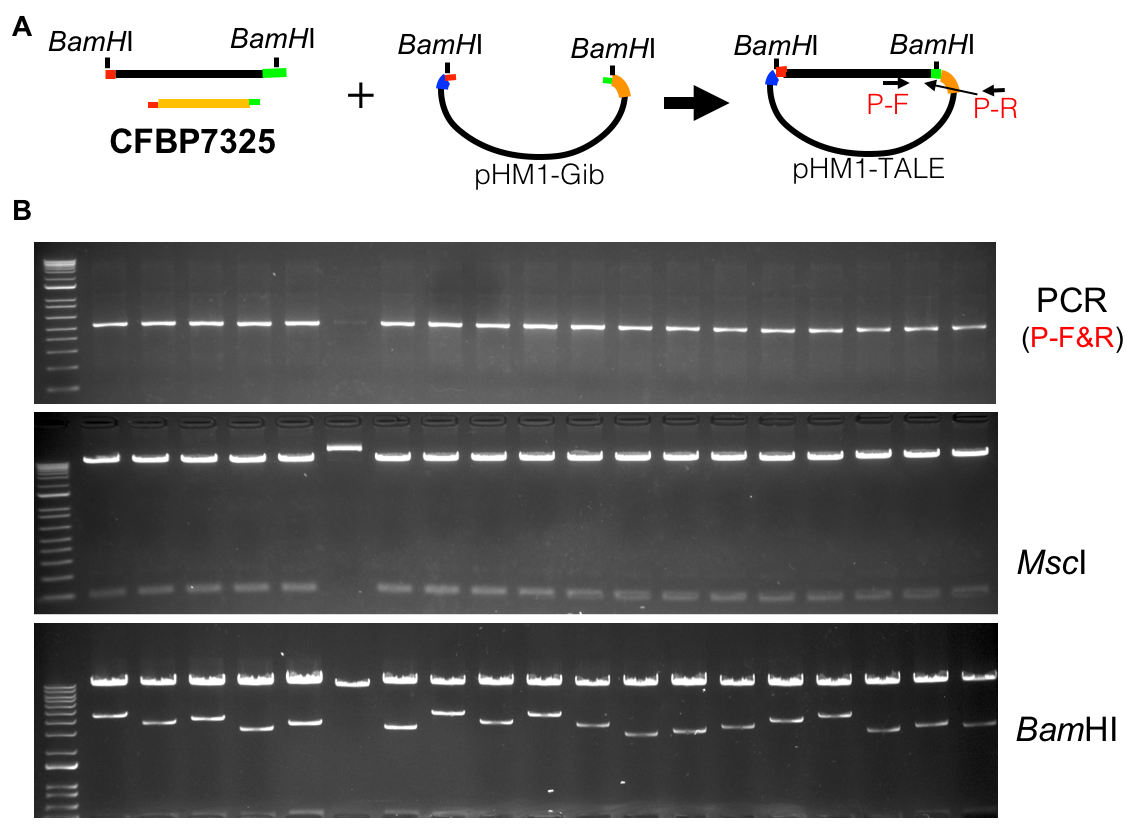
**

**Supplementary Fig. S6** Validation of TALe clones through PCR, restriction enzyme digestions. **A.** Schematics of selective isolation of *Bam*HI fragments TALe genes from genomic DNA of CFBP7325. **B.** Validation of TALe clones through PCR with primers P-F1 and P-R1 of individual clones as indicated above lanes of upper gel image, digestion by *Msc*I which cuts each of central repeats (the DNA band patterns resulted from partial digestion), and digestion by *Bam*HI.
