# Supplementary material for: An efficient method to clone TAL effector genes from Xanthomonas oryzae using Gibson assembly
Source: Mol Plant Pathol. 2019 Aug 15;20(10):1453–62. doi: 10.1111/mpp.12820 (PMC6792135; doi:10.1111/mpp.12820)
Supplement: Supplementary file 7 — Fig. S7 TALe gene distribution in two African Xoo genomes. (A, B) Nine TALe genes in each of two Xoo genomes are syntenic in their locations with the same colors denoting identical TALes at the amino acid level while different colors indicate different TALes at the amino acid level. [file MPP-20-1453-s007.docx]

**
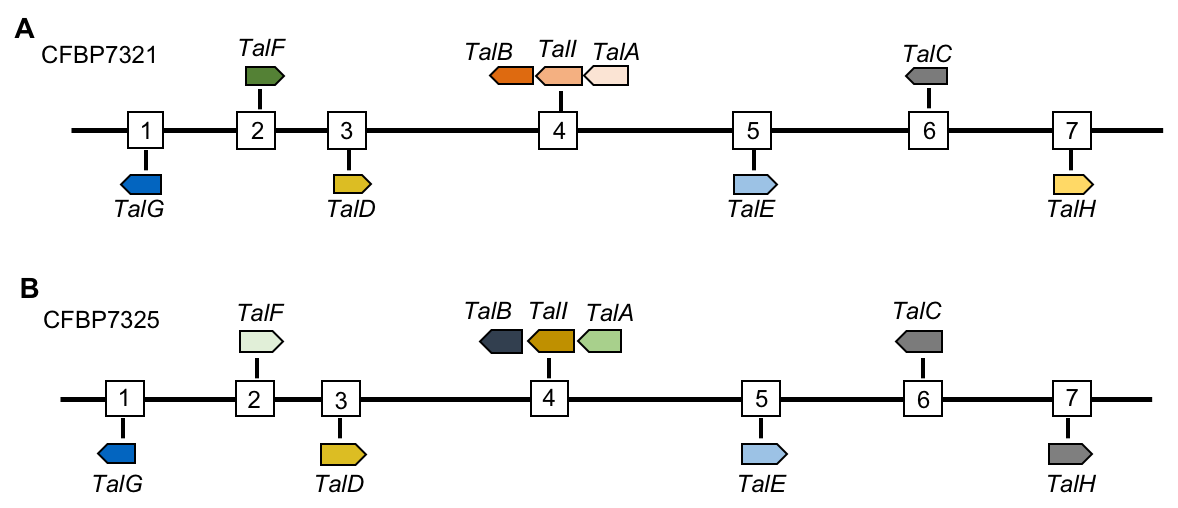
**

**Supplementary Fig. S7** TALe gene distribution in two African Xoo genomes. **A, B.** Nine TALe genes in each of two Xoo genomes are syntenic in their locations with same colors denoting identical TALes at the amino acid level while different colors indicating different TALes at the amino acid level.
