# Supplementary material for: An efficient method to clone TAL effector genes from Xanthomonas oryzae using Gibson assembly
Source: Mol Plant Pathol. 2019 Aug 15;20(10):1453–62. doi: 10.1111/mpp.12820 (PMC6792135; doi:10.1111/mpp.12820)
Supplement: Supplementary file 8 — Fig. S8 Two TALe genes cloned with the pHM1‐Gib system were functional in virulence. The virulences of TalC from CFBP7321 and TalF from CFBP7325 were tested in Kitaake leaves. Different letters indicate statistically significant differences. [file MPP-20-1453-s008.docx]

**
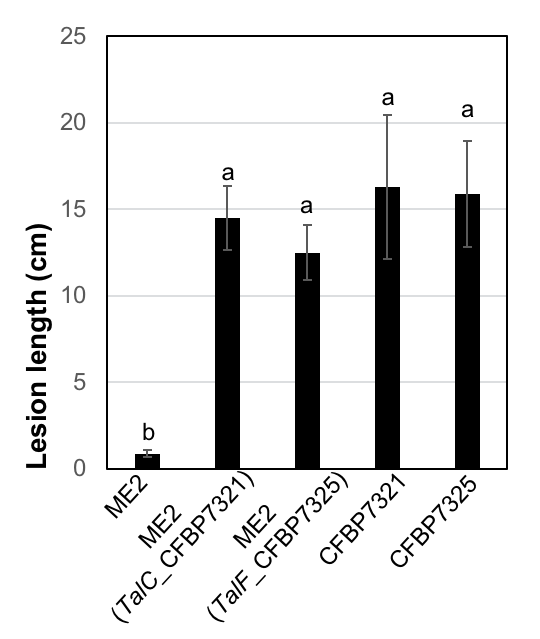
**

**Supplementary Fig. S8** Two TALe genes cloned with pHM1-Gib system were functional in virulence. The virulence of *TalC* from CFBP7321 and *TalF* from CFBP7325 were tested in Kitaake leaves. Different letters indicate statistically significant difference.
