# Supplementary material for: An efficient method to clone TAL effector genes from Xanthomonas oryzae using Gibson assembly
Source: Mol Plant Pathol. 2019 Aug 15;20(10):1453–62. doi: 10.1111/mpp.12820 (PMC6792135; doi:10.1111/mpp.12820)
Supplement: Supplementary file 9 — Table S1 Number of TALe genes from different Xanthomonas that can be selectively cloned with Gibson assembly. [file MPP-20-1453-s009.docx]

**Supplementary Table 1.** Number of TALe genes from different *Xanthomonas* that can be selectively cloned with Gibson assembly.

| ***Xanthomonas*** | | | **Number of TALe genes in genome** | **Number of TALe genes clonable as *Bam*HI-digested fragments** | **Number of TALe genes clonable as *Sph*I-digested fragments** |
| --- | --- | --- | --- | --- | --- |
| **Strain** | | **Accession No.** |  |  |  |
| ***X. o.* pv. oryzae**  (Asia) | **PXO61** | CP033187 | 18 | 17 | 17 |
|  | **XF89b** | GCA_002023005.1 | 17 | 17 | 17 |
|  | **PXO602** | GCA_001746735.1 | 20 | 19 | 20 |
|  | **PXO563** | GCA_001746715.1 | 18 | 17 | 17 |
|  | **PXO524** | GCA_001746695.1 | 19 | 17 | 18 |
|  | **PXO282** | GCA_001746675.1 | 18 | 17 | 17 |
|  | **PXO236** | GCA_001746655.1 | 16 | 14 | 15 |
|  | **PXO211** | GCA_001746635.1 | 17 | 15 | 16 |
|  | **PXO145** | GCA_001746615.1 | 18 | 15 | 17 |
|  | **PXO71** | GCA_001746595.1 | 20 | 17 | 18 |
|  | **PXO83** | GCA_001518895.1 | 18 | 15 | 17 |
|  | **PXO99^A^** | GCA_000019585.2 | 19 | 18 | 18 |
|  | **PXO86** | GCA_000948075.1 | 18 | 15 | 17 |
|  | **MAFF311018** | GCA_000010025.1 | 17 | 16 | 16 |
|  | **KACC10331** | AE013598.1 | 13 | 13 | 13 |
|  | **HuN37** | GCA_003382775.1 | 18 | 17 | 17 |
|  | **JL25** | GCA_003382795.1 | 16 | 15 | 15 |
|  | **JL28** | GCA_003382815.1 | 12 | 10 | 10 |
|  | **JL33** | GCA_003382835.1 | 16 | 15 | 15 |
|  | **JP01** | GCA_003382855.1 | 17 | 16 | 16 |
|  | **OS198** | GCA_003382875.1 | 18 | 18 | 18 |
|  | **YC11** | GCA_003382935.1 | 12 | 12 | 12 |
| ***X. o.* pv. oryzae**  (Africa) | **BAI3 (CFBP7321)** | GCA_003031385.1 | 9 | 9 | 9 |
|  | **MAI1 (CFBP7325)** | GCA_003031365.1 | 9 | 9 | 8 |
|  | **MAI145** | GCA_002850095.1 | 9 | 9 | 8 |
|  | **MAI134** | GCA_002850175.1 | 9 | 9 | 7 |
|  | **MAI129** | GCA_002850155.1 | 9 | 9 | 8 |
|  | **MAI106** | GCA_002850135.1 | 9 | 9 | 8 |
|  | **MAI99** | GCA_002850215.1 | 9 | 9 | 8 |
|  | **MAI95** | GCA_002850195.1 | 9 | 9 | 8 |
|  | **MAI73** | GCA_002850075.1 | 9 | 9 | 8 |
|  | **MAI68** | GCA_002850115.1 | 9 | 9 | 8 |
|  | **AXO1947** | GCA_001466505.1 | 9 | 9 | 9 |
| ***X. o.* pv. oryzicola** | **CFBP2286** | GCA_001042735.1 | 28 | 27 | 22 |
|  | **B8-12** | GCA_001042745.1 | 28 | 27 | 25 |
|  | **BLS256** | GCA_000168315.3 | 28 | 27 | 23 |
|  | **BLS279** | GCA_001042775.1 | 26 | 25 | 23 |
|  | **BXOR1** | GCA_001042795.1 | 25 | 24 | 25 |
|  | **CFBP7331** | GCA_001042815.1 | 20 | 20 | 19 |
|  | **CFBP7341** | GCA_001042835.1 | 20 | 19 | 18 |
|  | **CFBP7342** | GCA_000940825.1 | 23 | 23 | 21 |
|  | **L8** | GCA_001042855.1 | 29 | 28 | 26 |
|  | **RS105** | GCA_001042875.1 | 24 | 23 | 22 |
| ***X. citri* pv. vignicola** | **CFBP7112** | GCA_002218265.1 | 1 | 0 | 1 |
|  | **CFBP7113** | GCA_002218285.1 | 1 | 0 | 1 |
| ***X. citri* pv. malvacearum** | **XCMH1005** | GCA_002224525.1 | 6 | 6 | 5 |
| **Total TALe genes** | |  | 733 | 693 (94%) | 676 (92%) |
