# Supplementary material for: An efficient method to clone TAL effector genes from Xanthomonas oryzae using Gibson assembly
Source: Mol Plant Pathol. 2019 Aug 15;20(10):1453–62. doi: 10.1111/mpp.12820 (PMC6792135; doi:10.1111/mpp.12820)
Supplement: Supplementary file 10 — Table S2 Plasmids and bacterial strains used in this study. [file MPP-20-1453-s010.docx]

**Supplementary Table 2**. Plasmids and bacterial strains used in this study

| Designation | Genotypes or related characteristics | Source or reference |
| --- | --- | --- |
| Plasmids | |  |
| pHM1 | Broad host range, resistance to spectinomycin, *cos* site | (Hopkins *et al.*, 1992) |
| pZWpthXo1 | *Bam*HI fragment of *pthXo1* in pZW | (Yang & White, 2004) |
| pZW-Gib | *Sph*I fragment of gblock in pZWpthXo1 | This study |
| pZWpxo61tale1a | *SphI* fragment of *PXO61tale1a* in pZW-Gib | This study |
| pZWpxo61tale1b | *Sph*I fragment of *PXO61tale1b* in pZW-Gib | This study |
| pZWpxo61tale1c | *Sph*I fragment of *PXO61tale1c* in pZW-Gib | This study |
| pZWpxo61tale1d | *Sph*I fragment of *PXO61tale1d* in pZW-Gib | This study |
| pZWpxo61tale2a | SphI fragment of *PXO61tale2a* in pZW-Gib | This study |
| pZWpxo61tale2b | *Sph*I fragment of *PXO61tale2b* in pZW-Gib | This study |
| pZWpxo61tale2c | *Sph*I fragment of *PXO61tale2c* in pZW-Gib | This study |
| pZWpxo61tale3a | *Sph*I fragment of *PXO61tale3a* in pZW-Gib | This study |
| pZWpxo61tale3b | *Sph*I fragment of *PXO61tale3b* in pZW-Gib | This study |
| pZWpxo61tale4a | *Sph*I fragment of *PXO61tale4a* in pZW-Gib | This study |
| pZWpxo61tale4b | *Sph*I fragment of *PXO61tale4b* in pZW-Gib | This study |
| pZWpxo61tale4c | *Sph*I fragment of *PXO61tale4c* in pZW-Gib | This study |
| pZWpxo61tale5a | *Sph*I fragment of *PXO61tale5a* in pZW-Gib | This study |
| pZWpxo61tale6a | *Sph*I fragment of *PXO61tale6a* in pZW-Gib | This study |
| pZWpxo61tale6b | *Sph*I fragment of *PXO61tale6b* in pZW-Gib | This study |
| pZWpxo61tale6c | *Sph*I fragment of *PXO61tale6c* in pZW-Gib | This study |
| pZWpxo61tale7 | *Sph*I fragment of *PXO61tale7* in pZW-Gib | This study |
| pZWaxo1947tale1 | *Sph*I fragment of *AXO1947tale1* in pZW-Gib | This study |
| pZWaxo1947tale2 | *Sph*I fragment of *AXO1947tale2* in pZW-Gib | This study |
| pZWaxo1947tale3 | *Sph*I fragment of *AXO1947tale3* in pZW-Gib | This study |
| pZWaxo1947tale4a | *Sph*I fragment of *AXO1947tale4a* in pZW-Gib | This study |
| pZWaxo1947tale4b | *Sph*I fragment of *AXO1947tale4b* in pZW-Gib | This study |
| pZWaxo1947tale4c | *Sph*I fragment of *AXO1947tale4c* in pZW-Gib | This study |
| pZWaxo1947tale5 | *Sph*I fragment of *AXO1947tale5* in pZW-Gib | This study |
| pZWaxo1947tale6 | *Sph*I fragment of *AXO1947tale6* in pZW-Gib | This study |
| pZWaxo1947tale7 | *Sph*I fragment of *AXO1947tale7* in pZW-Gib | This study |
| pHM1-Gib | pHM1 vector with natural TALe’s promoter and colE1replication origin, *Bam*HI fragment of gblock | This study |
| pHM1-pZWpxo61tale1a | pZWpxo61tale1a in pHM1 | This study |
| pHM1-pZWpxo61tale1b | pZWpxo61tale1b in pHM1 | This study |
| pHM1-pZWpxo61tale1c | pZWpxo61tale1c in pHM1 | This study |
| pHM1-pZWpxo61tale1d | pZWpxo61tale1d in pHM1 | This study |
| pHM1-pZWpxo61tale2a | pZWpxo61tale2a in pHM1 | This study |
| pHM1-pZWpxo61tale2b | pZWpxo61tale2b in pHM1 | This study |
| pHM1-pZWpxo61tale2c | pZWpxo61tale2c in pHM1 | This study |
| pHM1-pZWpxo61tale3a | pZWpxo61tale3a in pHM1 | This study |
| pHM1-pZWpxo61tale3b | pZWpxo61tale3b in pHM1 | This study |
| pHM1-pZWpxo61tale4a | pZWpxo61tale4a in pHM1 | This study |
| pHM1-pZWpxo61tale4b | pZWpxo61tale4b in pHM1 | This study |
| pHM1-pZWpxo61tale4c | pZWpxo61tale4c in pHM1 | This study |
| pHM1-pZWpxo61tale5a | pZWpxo61tale5a in pHM1 | This study |
| pHM1-pZWpxo61tale6a | pZWpxo61tale6a in pHM1 | This study |
| pHM1-pZWpxo61tale6b | pZWpxo61tale6b in pHM1 | This study |
| pHM1-pZWpxo61tale6c | pZWpxo61tale6c in pHM1 | This study |
| pHM1-pZWpxo61tale7 | pZWpxo61tale7 in pHM1 | This study |
| pHM1-pZWaxo1947tale1 | pZWaxo1947tale1 in pHM1 | This study |
| pHM1-pZWaxo1947tale2 | pZWaxo1947tale2 in pHM1 | This study |
| pHM1-pZWaxo1947tale3 | pZWaxo1947tale3 in pHM1 | This study |
| pHM1-pZWaxo1947tale4a | pZWaxo1947tale4a in pHM1 | This study |
| pHM1-pZWaxo1947tale4b | pZWaxo1947tale4b in pHM1 | This study |
| pHM1-pZWaxo1947tale4c | pZWaxo1947tale4c in pHM1 | This study |
| pHM1-pZWaxo1947tale5 | pZWaxo1947tale5 in pHM1 | This study |
| pHM1-pZWaxo1947tale6 | pZWaxo1947tale6 in pHM1 | This study |
| pHM1-pZWaxo1947tale7 | pZWaxo1947tale7 in pHM1 | This study |
| pHM1-Gib-CFBP7321-TalC | BamHI fragment of *CFBP7321talc* in pHM1-Gib | This study |
| pHM1-Gib-CFBP7325-TalF | BamHI fragment of *CFBP7325talf* in pHM1-Gib | This study |
| Bacterial Strains |  |  |
| *Escherichia coli* |  |  |
| T1 | F-φ80(lacZ)ΔM15ΔlacX74hsdR(rk^-^, mk^+^)ΔrecA1398endA1tonA | Thermo Fisher Scientific |
| DB3.0 | Str^R^, *gyrA*462 *endA*1 Δ*(sr*1*-rec*A*) mcrB mrr hsdS*20 *glnV*44 *ara*14 *galK*2 *lac*Y1 *proA*2 *rps*L20 *xy*l5 *leuB*6 *mtl*1*,* resistant to ccdB | (Bernard & Couturier, 1992) |
| *Xanthomonas oryzae* pv. *oryzae* | |  |
| PXO99^A^ | Philippine race 6, azacytidine resistant clone of PXO99 | (Hopkins et al., 1992) |
| PXO61 | Philippine race 2 | (Yang & White, 2004) |
| AXO1947 | African strain | (Huguet-Tapia *et al.*, 2016) |
| ME2 | Mutant of PXO99^A^ with *pthXo1* inactivated | (Yang & White, 2004) |
| ME2(pHM1) | PXO99^A^ with pBY1 inserted into pthXo1 | (Yang & White, 2004) |
| ME2  (pHM1pZWpxo61tale1a) | ME2 with pHM1-pZWpxo61tale1a | This study |
| ME2(pHM1-pZWpxo61tale1b) | ME2 with pHM1-pZWpxo61tale1b | This study |
| ME2(pHM1-pZWpxo61tale1c) | ME2 with pHM1-pZWpxo61tale1c | This study |
| ME2(pHM1-pZWpxo61tale1d) | ME2 with pHM1-pZWpxo61tale1d | This study |
| ME2(pHM1-pZWpxo61tale2a) | ME2 with pHM1-pZWpxo61tale2a | This study |
| ME2(pHM1-pZWpxo61tale2b) | ME2 with pHM1-pZWpxo61tale2b | This study |
| ME2(pHM1-pZWpxo61tale2c) | ME2 with pHM1-pZWpxo61tale2c | This study |
| ME2(pHM1-pZWpxo61tale3a) | ME2 with pHM1-pZWpxo61tale3a | This study |
| ME2(pHM1-pZWpxo61tale3b) | ME2 with pHM1-pZWpxo61tale3b | This study |
| ME2(pHM1-pZWpxo61tale4a) | ME2 with pHM1-pZWpxo61tale4a | This study |
| ME2(pHM1-pZWpxo61tale4b) | ME2 with pHM1-pZWpxo61tale4b | This study |
| ME2(pHM1-pZWpxo61tale4c) | ME2 with pHM1-pZWpxo61tale4c | This study |
| ME2(pHM1-pZWpxo61tale5a) | ME2 with pHM1-pZWpxo61tale5a | This study |
| ME2(pHM1-pZWpxo61tale6a) | ME2 with pHM1-pZWpxo61tale6a | This study |
| ME2(pHM1-pZWpxo61tale6b) | ME2 with pHM1-pZWpxo61tale6b | This study |
| ME2(pHM1-pZWpxo61tale6c) | ME2 with pHM1-pZWpxo61tale6c | This study |
| ME2(pHM1-pZWpxo61tale7) | ME2 with pHM1-pZWpxo61tale7 | This study |
| ME2(pHM1-pZWaxo1947tale1) | ME2 with pHM1-pZWaxo1947tale1 | This study |
| ME2(pHM1-pZWaxo1947tale2) | ME2 with pHM1-pZWaxo1947tale2 | This study |
| ME2(pHM1-pZWaxo1947tale3) | ME2 with pHM1-pZWaxo1947tale3 | This study |
| ME2(pHM1-pZWaxo1947tale4a) | ME2 with pHM1-pZWaxo1947tale4a | This study |
| ME2(pHM1-pZWaxo1947tale4b) | ME2 with pHM1-pZWaxo1947tale4b | This study |
| ME2(pHM1-pZWaxo1947tale4c) | ME2 with pHM1-pZWaxo1947tale4c | This study |
| ME2(pHM1-pZWaxo1947tale5) | ME2 with pHM1-pZWaxo1947tale5 | This study |
| ME2(pHM1-pZWaxo1947tale6) | ME2 with pHM1-pZWaxo1947tale6 | This study |
| ME2(pHM1-pZWaxo1947tale7) | ME2 with pHM1-pZWaxo1947tale7 | This study |
| ME2(pHM1-CFBP7321-TalC) | ME2 with pHM1-CFBP7321-TalC | This study |
| ME2(pHM1-CFBP7325-TalF) | ME2 with pHM1-CFBP7325-TalF | This study |

**References**

Bernard, P. and Couturier, M. (1992) Cell killing by the F plasmid CcdB protein involves poisoning of DNA-topoisomerase II complexes. *J Mol Biol,* **226,** 735-745.

Hopkins, C. M., White, F. F., Choi, S. pZW-Gib., Guo, A. and Leach, J. E. (1992) Identification of a family of avirulence genes from *Xanthomonas oryzae* pv. oryzae. *Mol Plant Microbe Interact,* **5,** 451-459.

Huguet-Tapia, J. C., Peng, Z., Yang, B., Yin, Z., Liu, S. and White, F. F. (2016) Complete Genome Sequence of the African Strain AXO1947 of *Xanthomonas oryzae* pv. oryzae. *Genome Announc,* **4**,13435.

Yang, B. and White, F. F. (2004) Diverse members of the AvrBs3/PthA family of type III effectors are major virulence determinants in bacterial blight disease of rice. *Mol Plant Microbe Interact,* **17,** 1192-1200.
