# Supplementary material for: An efficient method to clone TAL effector genes from Xanthomonas oryzae using Gibson assembly
Source: Mol Plant Pathol. 2019 Aug 15;20(10):1453–62. doi: 10.1111/mpp.12820 (PMC6792135; doi:10.1111/mpp.12820)
Supplement: Supplementary file 11 — Table S3 Primers used in this study. [file MPP-20-1453-s011.docx]

**Supplementary Table 3.** Primers used in this study

| Name | Sequence (5’ to 3’) | Usage |
| --- | --- | --- |
| TalAatII-F | TTGGCCTGCCTCGGCGGACGTCCT | Amplify the 3’ region of TALe gene |
| TalFlH3-R | TGGCGGCCGCTCTAGGCCaagcttTCActtatcgtcatcgtccttgtaatcGATCGTCCCTCCGACTGAG |  |
| P-F | AGAGCATTGTTGCCCAGT | PCR screening clones for TALe genes |
| P-R | TCTGATCTCCCTCGTGCATTG |  |
| Tal-SphI-F | AGTTGGACACAGGCCAACTTC | Sequencing TALe repeat region from the 5’ end |
| P-R | TCTGATCTCCCTCGTGCATTG | Sequencing TALe repeat region from the 3’ end |
